# Supplementary material for: MTS1338, A Small Mycobacterium tuberculosis RNA, Regulates Transcriptional Shifts Consistent With Bacterial Adaptation for Entering Into Dormancy and Survival Within Host Macrophages
Source: Front Cell Infect Microbiol. 2019 Nov 26;9:405. doi: 10.3389/fcimb.2019.00405 (PMC6901956; doi:10.3389/fcimb.2019.00405)
Supplement: Supplementary Table 1 — Oligonucleotides used in the study. [file Table_1.DOCX]

**Supplementary Table 1. Oligonucleotides used in the study**

| Name | Sequence |
| --- | --- |
| MTS1338-f | ACCGGGGAAACCCGGTGAT |
| MTS1338-r | AACAGGATGAGGATCTGCCC |
| MTS1338 HindIII-f | TATTCGAAGGGGAAACCCGGTGATCT |
| MTS1338 HindIII-r | TATTCGAAAACAGGATGAGGATCTGCCC |
| qPCR_MTS1338-f | GTGCTGGGCGATTGAGC |
| qPCR_MTS1338-r | GCGGTAGCCCCGTCTT |
| qPCR_16S-f | TACGTAGGGTGCGAGCGTTG |
| qPCR_16S-r | CCCGCACGCTCACAGTTAAG |
| qPCR_Rv0081-f | GCCTGGAGTCGTCGAACCT |
| qPCR_Rv0081-r | GGGTGCGGCAATCGAATAGAT |
| qPCR_Rv0083-f | CGTTTCTGCTGGCGTGGGA |
| qPCR_Rv0083-r | CAACACCACCAGCCCGAC |
| qPCR_Rv0086-f | TTTGGGTAGCGATCGAGGCCA |
| qPCR_Rv0086-r | TACCCAAGAAGGCGACGGC |
| qPCR_Rv1621-f | GCTGGCTACCACTAACCCCTC |
| qPCR_Rv1621-r | TTCCGCGATGCGTTGTTCC |
| qPCR_Rv2986-f | CGTCACCATTACCGGGTTCG |
| qPCR_Rv2986-r | CACAACCGCTTTGAATTGCGC |
| qPCR_Rv3136-f | AAACAGCGATCCAAGCCAGG |
| qPCR_Rv3136-r | CCGCAGTGTTCTGGCCGA |
