# Supplementary material for: MTS1338, A Small Mycobacterium tuberculosis RNA, Regulates Transcriptional Shifts Consistent With Bacterial Adaptation for Entering Into Dormancy and Survival Within Host Macrophages
Source: Front Cell Infect Microbiol. 2019 Nov 26;9:405. doi: 10.3389/fcimb.2019.00405 (PMC6901956; doi:10.3389/fcimb.2019.00405)
Supplement: Supplementary Figure 1 — (A) MTS1338 transcription level in samples used for RNA-seq. pMV stands for control M. tuberculosis strains. Cl2, cl8, cl4—OVER strains, independent clones. (B) Validation of RNA-seq data by qRT-PCR. [file Data_Sheet_1.docx]

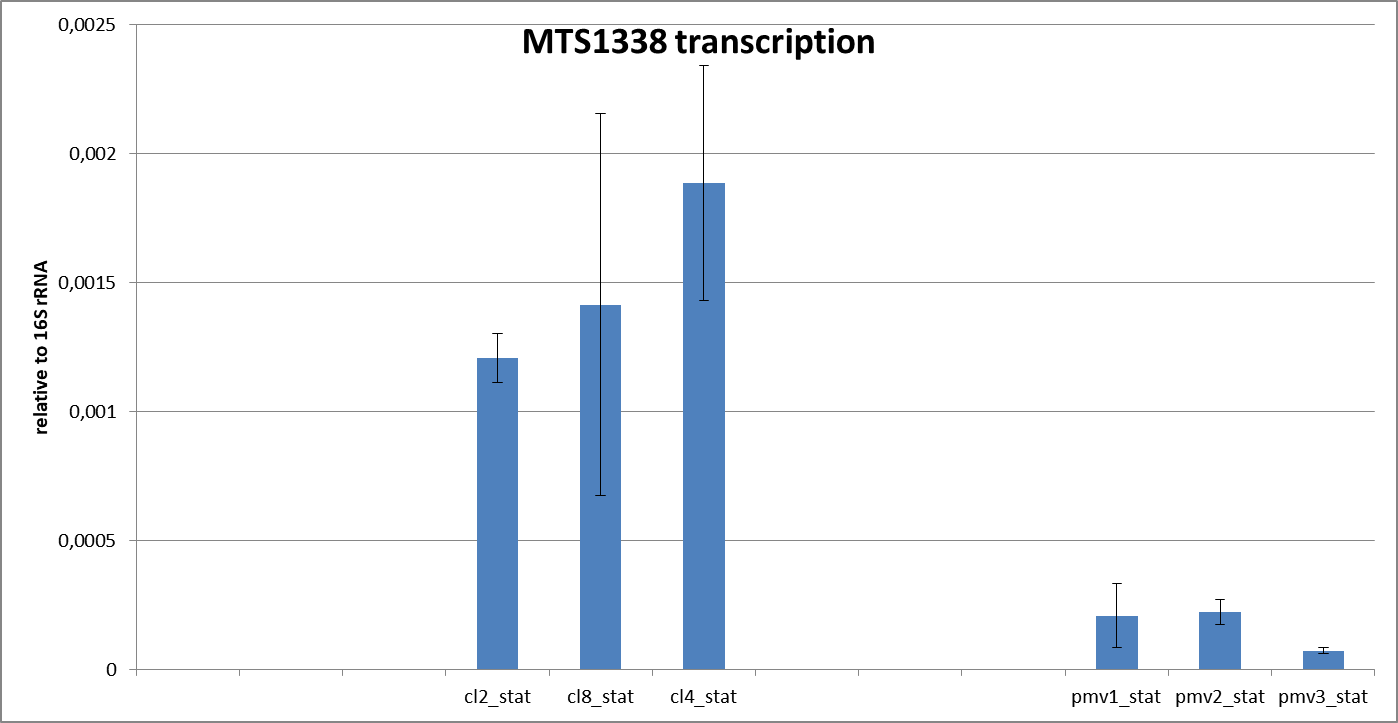


Figure 1A. MTS1338 transcription level in samples used for RNA-seq. pMV stands for control *M.tuberculosis* strains. Cl2, cl8, cl4 – OVER strains, independent clones.


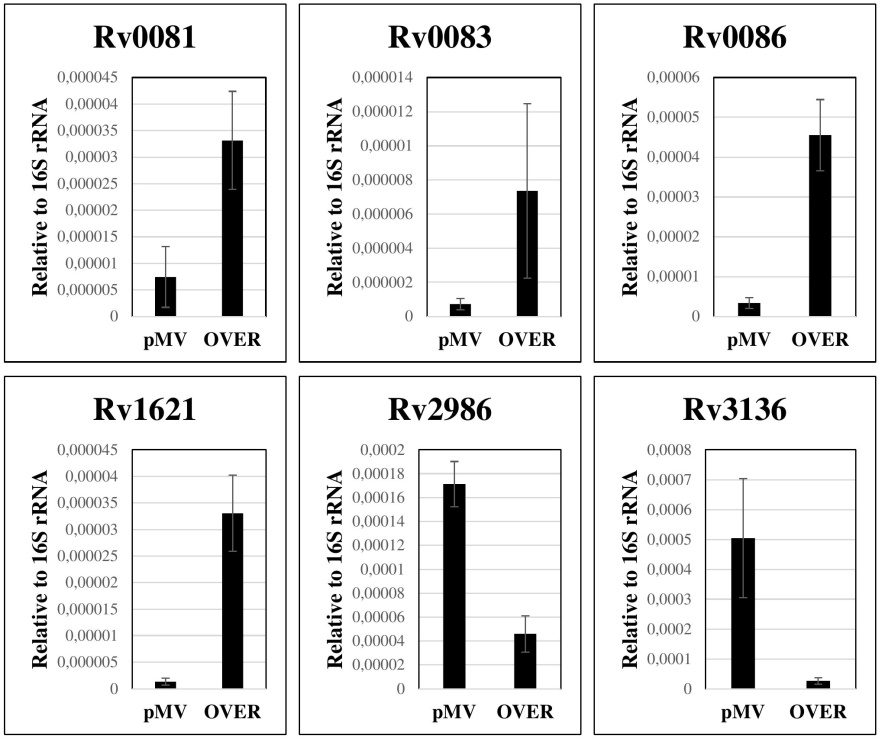


Figure 1B. Validation of RNA-seq data by qRT-PCR
